# Computational Saturation Mutagenesis to Investigate the Effects of Neurexin-1 Mutations on AlphaFold Structure

**DOI:** 10.3390/genes13050789

**Published:** 2022-04-28

**Authors:** Raina Rhoades, Brianna Henry, Dominique Prichett, Yayin Fang, Shaolei Teng

**Affiliations:** 1 Department of Biology, Howard University, Washington, DC 20059, USA; raina.rhoades@bison.howard.edu (R.R.); brianna.henry@bison.howard.edu (B.H.); dominique.pritchett@howard.edu (D.P.); 2 Department of Biochemistry and Molecular Biology, Howard University, Washington, DC 20059, USA; yfang@howard.edu

**Keywords:** neurexin-1, AlphaFold, missense mutation, protein stability, computational saturation mutagenesis

## Abstract

Neurexin-1 (NRXN1) is a membrane protein essential in synapse formation and cell signaling as a cell-adhesion molecule and cell-surface receptor. NRXN1 and its binding partner neuroligin have been associated with deficits in cognition. Recent genetics research has linked NRXN1 missense mutations to increased risk for brain disorders, including schizophrenia (SCZ) and autism spectrum disorder (ASD). Investigation of the structure–function relationship in NRXN1 has proven difficult due to a lack of the experimental full-length membrane protein structure. AlphaFold, a deep learning-based predictor, succeeds in high-quality protein structure prediction and offers a solution for membrane protein model construction. In the study, we applied a computational saturation mutagenesis method to analyze the systemic effects of missense mutations on protein functions in a human NRXN1 structure predicted from AlphaFold and an experimental *Bos taurus* structure. The folding energy changes were calculated to estimate the effects of the 29,540 mutations of AlphaFold model on protein stability. The comparative study on the experimental and computationally predicted structures shows that these energy changes are highly correlated, demonstrating the reliability of the AlphaFold structure for the downstream bioinformatics analysis. The energy calculation revealed that some target mutations associated with SCZ and ASD could make the protein unstable. The study can provide helpful information for characterizing the disease-causing mutations and elucidating the molecular mechanisms by which the variations cause SCZ and ASD. This methodology could provide the bioinformatics protocol to investigate the effects of target mutations on multiple AlphaFold structures.

## 1. Introduction

Resolving protein structures experimentally can be achieved by using X-ray crystallography, NMR spectroscopy, electron microscopy, and electron diffraction. Although technological advancements in software and hardware have improved resolution, these techniques can be extremely challenging, time-consuming, and costly. Hundreds of millions of proteins are listed in the UniProt database, but a relatively small number are represented in the Protein Data Bank (PDB) [1]. Thus, having reliable computational modeling approaches to investigate proteins is vital for understanding structure and function, protein–protein interactions, and drug discovery. The latest tool, called AlphaFold [2], has proved to be the most accurate, demonstrating a median backbone root mean squared deviation (RMSD) of 0.96 Å [3]. AlphaFold uses the deep neural network algorithm that utilizes homologous templates and multiple sequence alignment to predict protein structures. The initial inputs to the algorithm are the primary protein sequence of interest and homologous proteins, which are used to construct a multiple sequence alignment [3]. Having access to better models will make it possible to investigate the molecular properties of proteins within an evolutionary context, as we can compare AlphaFold structures across multiple species. The highly accurate models predicted by the AlphaFold make it an important tool that can be used to investigate the structure and function of proteins for which we lack experimentally derived structures, such as neurexin-1 (NRXN-1).

Neurexins are a group of polymorphic cell adhesion molecules that are important in neurotransmission and synaptogenesis [4]. These proteins are primarily expressed in presynaptic terminals and can bind several soluble and postsynaptic proteins [5]. Mammals have three neurexin genes, which possess two different promoters capable of transcribing longer α isoforms and shorter β isoforms [5,6,7]. Several splice variants are known for both α and β isoforms, five splice sites are known for α neurexin, and two splice sites are known to occur in β neurexin. NRXN1a is expressed in many brain regions, including the cerebellum, claustrum, and thalamus [8]. Several complex mental disorders are associated with mutations in NRXN1, along with dysfunction in the cerebellum, including ASD, SCZ, and intellectual disability [5,9,10,11,12]. While overlapping expression patterns have been demonstrated for NRXN1, NRXN2, and NRXN3 with some neurons expressing multiple neurexins, the expression of these proteins is essential as they help determine the balance of the excitatory/inhibitory properties of synapses based on the proteins they are capable of binding [8,13,14,15]. The expression of NRXN1 in the brain is regulated by the Disrupted in Schizophrenia 1 protein (DISC1). Previous investigations of RNA expression in an SCZ mouse model carrying a missense mutation in Disc1 demonstrated dysregulated Nrxn1 and Nrxn3 expression profiles, indicating that Disc1 plays a role in regulating Nrxn1 [16]. Previous work demonstrated that NRXN1 expression increases significantly with age in humans. Neurexins are known to bind extracellularly with neuroligin, dystroglycan, neurexophilins, and leucine-rich repeat transmembrane neuronal protein (LRRTM) [17].

The NRXN1α protein can be up to 1477 amino acids in length. The domain structure of NRXN1 consists of a large extracellular domain with an N-terminal signal peptide, three epidermal growth factor-like (EGF-like) domains, each of which is flanked by two of the six laminin neurexin sex hormone-binding globulin (LNS) domains, and this is followed by an O-glycosylated domain that connects the extracellular domains to the transmembrane region. Finally, a protein 4.1 binding site and a postsynaptic density zone (PDZ) domain binding site 129–131⁠ are found at the C terminus. The protein has an overall L shape with two hinge regions [18]. However, the β isoform possesses only a single extracellular LNS domain. The LNS domain structure is made up of a “lectin-like β-sheet with a conserved Ca^2+^” binding-site [19,20,21]. The NRXN1 protein is subject to extensive alternative splicing, leading to the potential generation of greater than 2000 variants [17]. These isoforms (NRXN1a and NRXN1b) and their respective variants seem to play different roles within the synapse [6]. For example, deletion of α neurexins results in diminished vesicle release from presynaptic terminals in response to the induction of action potentials and altered Ca+ channel function [22,23,24]; α neurexins are also crucial to N-methyl-D-aspartate (NMDA) receptor function, as a-neurexin knockout mice demonstrated a fifty percent decrease in the ratio of evoked synaptic currents between NMDARs to AMPARs [25]. One major binding partner of both α and β neurexins is neuroligin; these proteins bind postsynaptic density zone scaffolding proteins. Neuroligins recruit postsynaptic proteins such as receptors and ion channels and are important for synapse maturation and in the assembly of the postsynaptic density (PSD) proteins [26,27,28,29,30].

The aim of the current study is to demonstrate the utility of AlphaFold for the study of the evolution of proteins such as NRXN1. We compared the sequence and structural similarity between the experimentally derived *Bos taurus* structure (PDB ID: 3poy) and the computationally predicted human AlphaFold model (Uniprot ID: Q9ULB1). To determine the effect of mutations on protein stability, we performed saturation mutagenesis on the experimentally determined and predicted NRXN1 structures and calculated the difference in free folding energy between the wildtype and mutated structures. The stability changes upon NRXN1 mutations were compared to deleterious effects for predicting the damaging missense mutations. We investigated the effects of known disease-causing mutations on NRXN1 stability and function. Finally, we examined the stability effects in the context of evolutionary conservation.

## 2. Materials and Methods

### 2.1. Structure Preparation

We collected the AlphaFold structure for human NRXN1a (UniProt ID: Q9ULB1) from the AlphaFold database [2]. The experimentally derived structures were downloaded from the Protein Data Bank (PDB). We also collected a homology model from the SWISS-MODEL Repository, 3qcw.1.A [31]. One of the largest NRXN1 structures available is the crystal structure of the α-Neurexin-1 ectodomain, LNS 2–6 from *B.taurus* (Bovine) (PDB ID: 3poy) [18]. The sequence of the experimental 3poy structure is 95.8% identical to the canonical sequence of the human NRXN1 protein, based on pairwise alignment [32,33]. The 3poy structure includes two EGF-like domains and five of the six laminin-neurexin-sex-hormone binding globulin domains, terminating just before the O-glycosylated stalk region [18]. We applied Pymol to compare the AlphaFold and experimental NRXN1 structures using the “super” algorithm, which uses a sequence-independent approach to align two structures that subsequently undergo a series of refinement cycles until the best fit is achieved [34].

### 2.2. Mutation Collection

To investigate the effect of mutations on protein stability in the NRXN1, we utilized the computational saturation mutagenesis procedure described in our recent study [35]. In brief, we generated all possible mutations in the structure by mutating each residue to each of the 19 other common amino acids. Additionally, the disease-causing mutations were collected using the Human Gene Mutation Database (HGMD) [36].

### 2.3. Sequence-Based Analysis

A pairwise global alignment was performed using EMBOSS NEEDLE for the human (Q9ULB1) and bovine (Q28146) NRXN1 sequences [32,33].

Transmembrane helices were predicted using bioinformatics tools. The TMHMM is a transmembrane hidden Markov model prediction tool that uses protein sequence data to predict transmembrane regions [37,38]. The DeepTMHMM is a protein structure prediction tool that uses a deep learning algorithm to predict transmembrane domains and is meant to replace the TMHMM tool [39]. The TOPCONS web server is a protein topology prediction tool that generates a prediction based on the consensus of the output from several protein structure prediction tools [40].

We utilized the sequence-based machine learning approach, SNAP2 [41], to obtain the damaging scores to predict the mutation functional effects. The SNAP2 tool is a classifier that uses neural network-based algorithms to determine whether a particular mutation is likely to be neutral or cause an effect on protein function.

### 2.4. Structure-Based Stability Calculation

We utilized Foldx version 5 to calculate changes in protein stability in the NRXN1a structures we collected. Foldx was used to derive information concerning the total folding energy (ΔΔG) and the contribution of entropy, hydrogen bonding, van der Waals interactions, and electrostatic interactions [42]. We first used Foldx to repair structures using the ”RepairPDB” command, which optimizes the structure by adjusting van der Waal’s interactions, torsion angles, etc. Using the command-line interface, we generated mutations by converting each residue to each of the 19 remaining amino acids. Then, we used Foldx to calculate the free energy, ΔG, for both the wildtype and the mutant structures, using the function ”BuildModel” [42]. The folding energy change upon mutation was calculated using the following equation:

ΔΔG_(stability)_ = ΔG_(folding)MUT_ − ΔG_(folding)WT_

The ΔΔG_(stability)_ scores can be used to estimate the effects of missense mutation on protein stability, whereby negative scores represent increased stability and positive scores represent decreased stability. These scores are grouped into five categories: highly stabilizing (ΔΔG < −2.0 kcal/mol), moderately stabilizing (−2.0 < ΔΔG < −0.5 kcal/mol), neutral (0.5 < ΔΔG < +0.5 kcal/mol), moderately destabilizing (+0.5 < ΔΔG < 2.0 kcal/mol), and highly destabilizing (ΔΔG > 2.0 kcal/mol).

We calculated the correlational coefficient for alanine mutations, all mutations, and the mean value of ΔΔG of mutations generated in both the bovine experimental and human AlphaFold structures by using the formula:

$$r=\sum \left(xi-x\right)\left(yi-y\right)/\surd \left[\sum \;\left(xi-\hat{x}\right)2\right]\left[\sum \;\left(yi-\hat{y}\right)2\right]$$

### 2.5. Conservation Analysis

Conservation scores were obtained from Consurf and Aminode tools [43,44]. Consurf uses user-provided amino acid sequences to find homologous sequences using BLAST. A list of the twenty species used in this study can be found in Supplementary Figure S1. The program then performs a multiple sequence alignment to the homologous sequences using a specified algorithm. The resulting alignment is used to build a phylogenetic tree which is then used to calculate “position-specific” conservation scores [43]. The conservation scores are converted to a color scale of 1–9, where highly variable residues are at the low end of the scale and those that are highly conserved are at the high end of the scale. Aminode is a bioinformatics tool based on “Amino Acid Evolutionary Constrained Analysis”. The Aminode tool utilizes amino acid sequences and phylogenetic trees to determine the best fit according to a “maximum parsimony approach”. The program then calculates a substitution score, whereby the higher the score, the rarer the likelihood of amino acid substitution [44]. The scores are then normalized across the entire MSA, which includes 23 species (Supplementary Figure S1). Consurf uses user-provided amino acid sequences to find homologous sequences using BLAST. The program then performs a multiple sequence alignment to the homologous sequences using a specified algorithm. The same species used in the Aminode analysis were used for the Consurf analysis. 

## 3. Results

### 3.1. Comparison of AlphaFold with Other Structures

**Figure 1:**
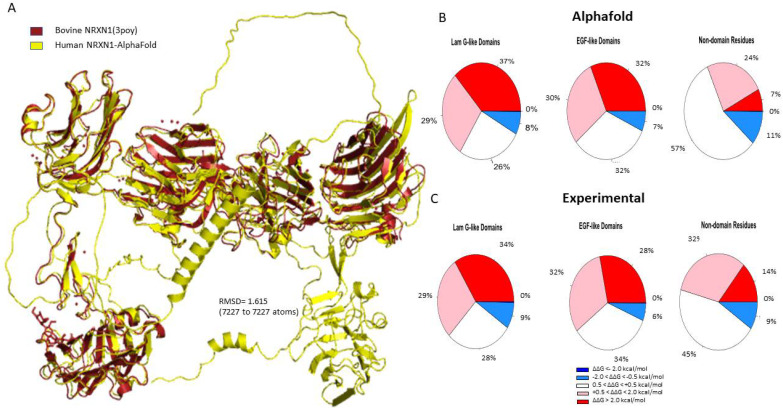
(**A**) Structural alignment of the human AlphaFold NRXN1 model. (**B**,**C**) Pie charts depicting the percentages concerning stability changes calculated following saturation mutagenesis for AlphaFold NRXN1 and experimental NRXN1 structures. The ΔΔG values are separated into highly destabilizing (dark red), moderately stabilizing (pink), neutral, moderately stabilizing (light blue), highly stabilizing (dark blue).

The pairwise sequence alignment of the human AlphaFold model and the experimental bovine structure (3poy) resulted in a percent identity of 95.8% [33]. The human NRXN1 AlphaFold structure aligns very closely with the experimental structure (RMSD = 1.615 based on 7227 atoms) (Figure 1A). To further evaluate the reliability of AlphaFold model, we compared the AlphaFold and experimental NRXN1 models with the homology model obtained from the SWISS-MODEL Repository (3qcw.1.A). As shown in Supplementary Figure S2B, the RMSD value between the AlphaFold NRXN1 to the SWISS-MODEL NRXN1 is 1.944 (7325 to 7325 atoms). When we made a similar comparison between the experimentally derived bovine structure and the SWISS-MODEL, we found an RMSD value of 0.954 (5855 to 5855 atoms) (Supplementary Figure S2A). The RMSD of the structural comparison of the experimental and SWISS-MODEL structures is lower than that obtained for the AlphaFold. The reason is that the AlphaFold model has all 6 LNS domains along with the transmembrane domain and C terminal domain, which are not modeled in homology modeling or experimental approaches.

The full-length human NRXN1 AlphaFold structure is 1477 residues in length. Thus, we generated 29,540 nonredundant missense mutations, which were used to calculate overall protein stability. The stability heatmaps for the AlphaFold and bovine NRXN1 models are displayed in Supplementary Figure S3. The heatmaps appear very similar except for the transmembrane domain and C terminus, which are present in the AlphaFold model but absent in the experimental structure. We specifically calculated the percentages of stabilizing and destabilizing mutations in each domain of the NRXN1 proteins.

**Figure 2:**
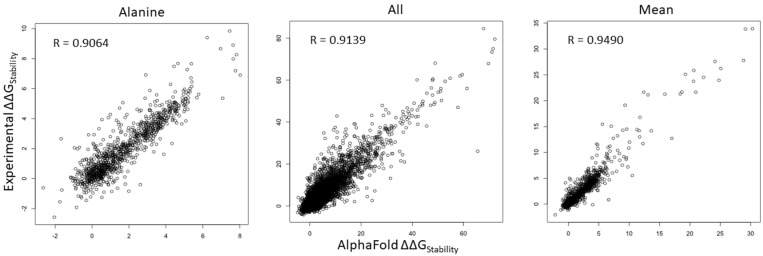
Scatterplots depicting the corrections of ΔΔG of all mutations (left), ΔΔG of substitutions to alanine (middle), and ΔΔG mean of residues (right) between the AlphaFold and experimental structures.

We compared ΔΔG values of all mutations in all of the positions within the human NRXN1 AlphaFold model and experimental structure. As shown in Figure 2, Pearson’s correlation coefficient (R) value of ΔΔG is 0.9064 for substitutions to alanine (A) and increases to 0.9139 for all mutations. We checked the substitutions to different residues and found that the *R*-values ranged from 0.8649 for substitutions to proline (P) to 0.9337 for substitutions to aspartic acid (D) (Supplementary Figure S4). Interestingly, the *R*-value is improved to 0.9490 for mean residue ΔΔG values (Figure 2). These results indicate that saturation mutagenesis of residues common to the bovine experimental and human AlphaFold NRXN1 models results in similar changes to the overall folding energy of the protein structure. The significant positive correlation of residue mean ΔΔG value (*R* = 0.9490, *p* < 2.2 × 10^−16^) implies that the mean ΔΔG is a reliable measure for predicting the effects of mutations in key residues on protein stability from the AlphaFold model.

### 3.2. Laminin G-like Domains and EGF Domains

There are six laminin G-like domains in the full-length NRXN1a protein; however, only the last laminin G-like domain (LNS6) is incorporated into the NRXN1b isoform. Most of the mutations generated in the Laminin G-like domains of the AlphaFold NRXN1 structure were destabilizing (37% highly destabilizing, 29% moderately destabilizing). Of the remaining residues, 26% were found to be neutral and 8% were found to be moderately stabilizing to the protein structure (Figure 1B). Four of the most destabilizing mutations, G587W, G850W, G1256, and G182, and all five of the top stabilizing mutations, were found in Laminin G-like domains.

Sixty-two percent of the mutations we generated in the EGF-like domains of the NRXN1 protein were found to be moderately to highly destabilizing (32% highly destabilizing, 30% moderately destabilizing). Seven percent of mutations generated in the EGF-like domains were moderately stabilizing. Mutations generated in the remaining residues were neutral in their effect on stability (32%) (Figure 1B).

**Figure 3:**
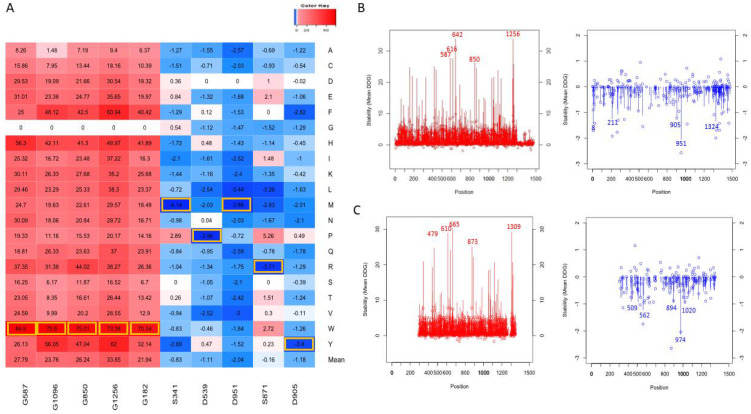
Top mutations affecting the protein stability (**A**). Top mutations are highlighted with yellow boxes. The line graph represents the mean ΔΔG (line) and ΔΔG of alanine mutations (shown as bubbles) for each position in the AlphaFold NRXN1 (**B**) and experimental bovine NRXN1 (**C**) structures.

Of the three EGF-like domains, the EGF-like 2 domain has the most significant percentage of mutations predicted to be moderately to highly destabilizing. It is worth noting that the EGF-like 2 domain, which is found between positions 676 and 713 and is a highly conserved domain (Evalue = 2.98e − 07) (Supplementary Figure S3) [45]. Based on proteins that contain homologous EGF-like domains, this region may possess an aspartate/asparagine hydroxylation site between positions 691 and 702 (Interpro/Prosite) (Supplementary Figure S5). The consensus sequence for this PTM is C-x- [DN]-x(4)- [FY]-x-C-x-C, and the sequence in the canonical sequence of human NRXN1 is C-R-D-G-W-N-R-Y-V-C-D-C. Though the consensus sequence does not completely match the sequence in the human NRXN1 EGF domain, the positions 691–702 may play an essential role in maintaining the stability of the overall protein as the average ΔΔG for these positions are 3.21, 1.572, 0.768, 6.778, 2.617, 2.363, 1.713, 1.181, and 0.841 kcal/mol, respectively. It is noteworthy that one of the most destabilizing mutations we found in the full-length AlphaFold NRXN1 structure, G1096W, is found in the EGF-like 3 domain (Figure 3).

We compared the ΔΔG of the mutations we generated by domain using a one-way ANOVA and found there were significant differences between the domains (F = 16.39, *p* < 2e − 16). The results of the Tukey test are displayed in Supplementary Table S1. Nondomain positions were found to be significantly different from positions located within domain regions (*p* < 0.01). The mean of the ΔΔG for non-domain positions in the NRXN1 protein is 0.49 kcal/mol (SD = 1.787), indicating that mutations in these residues are likely to result in a neutral change in overall stability. The mean ΔΔG for mutations occurring in residues contained in the recognized domains was 2.802 kcal/mol (SD = 5.102), indicating that the generation of missense mutations in the recognized domains is likely to cause destabilization in the NRXN1 protein.

### 3.3. Top Mutations Affecting the Protein Stability

As shown in Figure 3A for the AlphaFold model, the line graph represents the distribution of mean ΔΔG values, red represents positive values for the average destabilization effects of the NRXN1 protein upon mutation, and blue represents for negative values the average stabilization effects of the mutations in the residue positions. The top five most destabilizing positions in terms of average ΔΔG were 642, 1256, 587, 616, and 850 (ΔΔG < −1.1 kcal/mol). The top five most stabilizing positions in terms of average ΔΔG were 951, 8, 1324, 211, and 905 (ΔΔG > 25 kcal/mol). The bubbles represent the ΔΔG values of substitutions to alanine at each position. Note that the mean ΔΔG values and ΔΔG values of substitutions to alanine for experimental structure (Figure 3B) have a similar distribution to those for the AlphaFold model (Figure 3A).

**Figure 4:**
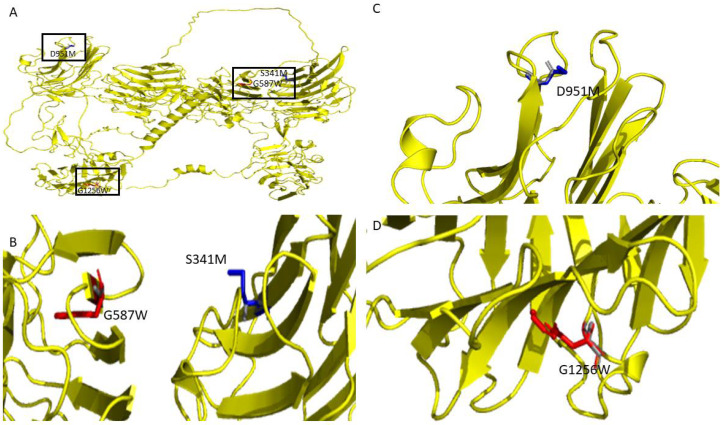
(**A**) Graphical depiction of selected top mutations from the analysis of the NRXN1 AlphaFold structure. Structural representations of (**B**) G587W, S341M, (**C**) D951, and (**D**) G1296.

Figure 3C depicts the top mutations that have destabilizing and stabilizing effects based on their ΔΔG values on the overall protein stability of the human NRXN1. The top five stabilizing mutations were D951M, S871R, D951L, D539P, and S341M (ΔΔG < −3.4 kcal/mol). The most destabilizing mutations were G182W, G1256W, G850W, G1096W, and G587W (ΔΔG > 70 kcal/mol). The top destabilizing alanine mutations are G587A, G417A, G616A, G1256A, and G642A (ΔΔG > 8.2 kcal/mol). The top stabilizing alanine mutations are L265A, S720A, S211A, T1324A, and D951A (ΔΔG < −1.701 kcal/mol). The top stabilizing and destabilizing mutations G587W, G1256W, S341M, and D951M are depicted in Figure 4B.

The top destabilizing mutations in the full-length experimental structure (3poy) were G610W, G665W, G1309W, G873W, and G1119W. It is worth noting that the G610W, G665W. G1309W, G873W, and G1119W are equivalent to the G587W, G642W, G1256W, G850W, and G1096W mutations we generated in the NRXN1 AlphaFold model based on pairwise alignment (Figure 3A,B). These and other similarities could be important in understanding the evolutionary history of the NRXN1 protein.

### 3.4. Transmembrane Region Prediction

**Figure 5:**
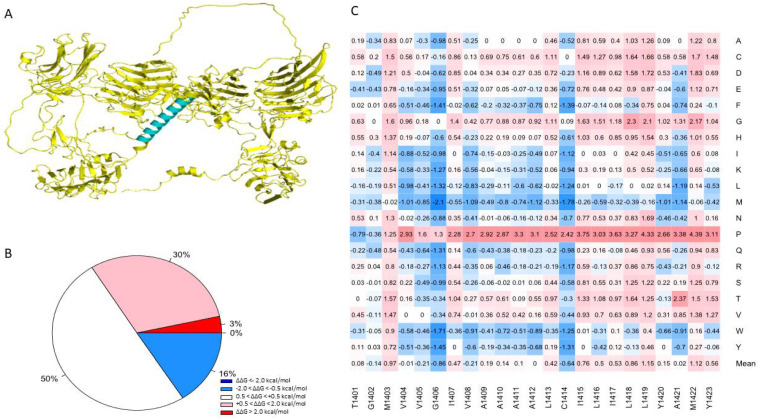
(**A**) Illustration of the transmembrane domain of the NRXN1 protein. (**B**) Piechart shows the percentages of predicted ΔΔG values. (**C**) Heatmap of residues 1401–1423, which correspond to the transmembrane domain. The color gradient corresponds to the degree of stability change: dark red to light red indicates highly to moderately destabilizing mutations, white indicates neutral, and dark blue to light blue indicates highly to moderately stabilizing mutations.

One advantage of using the human NRXN1 AlphaFold model is that it predicts the transmembrane domain lacking in the bovine experimental model. TMHMM predicts a structure consisting of an extracellular portion consisting of a signal peptide of 1–1400 amino acids, a transmembrane region with 22 amino acids in length, and an intracellular portion of 53 amino acids in length. TOPCONS produced a similar prediction; however, it predicted that positions 1–30 act as a signal peptide (Supplementary Figure S6). Most of the mutations in the region predicted by TMHMM to contain the transmembrane helix were neutral in terms of their effect on stability (50.2%), with an overall mean of 0.256 (Figure 5B). Based on the MSA performed by Consurf, this region is highly conserved among humans and 22 other species (Supplementary Figure S1). Sixteen percent of mutations generated in this region were predicted to moderately increase the overall stability of NRXN1. The remaining mutations were moderately (30.4%) or highly destabilizing (3.4%) (Figure 5A). Using the sequence corresponding to the transmembrane region, we used the lipidation prediction tool, CSS-Palm, and found an S-palmitoylation site at a cysteine residue at position 1414 [46]. The average ΔΔG of mutations located at position 1414 is −0.639 kcal/mol and therefore generally predicted to increase protein stability, with one notable exception being C1414P, which resulted in a ΔΔG of 2.417 kcal/mol, indicating a moderate decrease in protein stability. This mutation has not been reported but could be significant in understanding the potential role of S-palmitoylation or other post-translational modifications in the NRXN1 protein.

### 3.5. Mutation Pathogenicity Prediction

We evaluated the damage scores obtained for the full-length AlphaFold NRXN1 model using SNAP2. The correlational analysis found a moderate yet significant association between the protein stability and the SNAP2 scores (r = 0.334, *p* < 2.2 × 10^−16^). There was a significant difference in stability between the mutations predicted to cause and “effect” and those predicted to be “neutral” (t = −55.576, df = 21680, *p*-value < 2.2 × 10^−16^) (Supplementary Figure S7). We then compared SNAP2 scores based on whether their stability scores were predicted to be highly destabilizing to highly stabilizing and found a significant difference between groups (one-way ANOVA, df = 3, F = 2649, Pr < 2.2 × 10^−16^). The Tukey test demonstrated significant differences between all groups except the moderately stabilizing and moderately destabilizing groups (*p* < 0.1).

### 3.6. Disease-Causing Mutations

**Figure 6:**
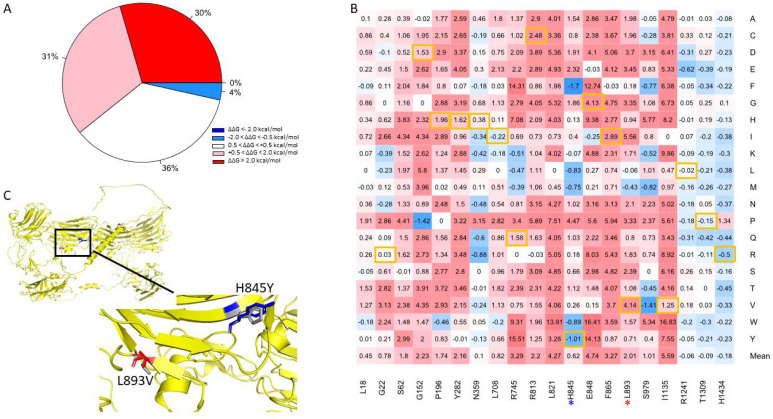
(**A**) Pie chart of the mutations in positions associated with disease-causing mutations. (**B**) Heatmap of mutations in positions associated with disease-causing variants. The color gradient corresponds to the degree of stability change: dark red to light red indicates highly to moderately destabilizing mutations, white indicates neutral, and dark blue to light blue indicates highly to moderately stabilizing mutations. Disease-causing mutations are highlighted in yellow. (**C**) Illustration of two top mutations: H845Y and L893V associated with ASD.

We attempted to verify the positions of the mutations by comparing the canonical sequence of human NRXN1 with those referenced in the papers from which the disease-causing mutations were derived using pairwise alignment (Supplementary Table S2.). We were able to validate 21 of the 24 mutations we obtained from HGMD [36]. Of the 400 mutations generated at positions corresponding to known disease variants, we found that 61% were predicted to be moderately to highly destabilizing (Figure 6A). Approximately 4% of mutations generated were found to be moderately stabilizing, while 36% of the mutations were found to be neutral. The residue I1135V is located in a position that is associated with the greatest mean destabilization of the NRXN1 protein (Mean ΔΔG = 5.59 kcal/mol) (Figure 6B). A mutation in this location, I1135V, is associated with ASD and results in moderate destabilization of the overall protein (ΔΔG = 1.25 kcal/mol). The most stabilizing position associated with disease-causing mutation is H1434. Mutations generated in this position have a mean ΔΔG of −0.18 kcal/mol. A mutation in this position, H1434R, is associated with SCZ and has ΔΔG of −0.5 kcal/mol. Of all the disease-causing mutations, the L893V mutation associated with ASD was the most destabilizing, with a ΔΔG of 4.14 kcal/mol (Figure 6C). Interestingly, the most stabilizing disease-causing mutation is H845Y, which is also associated with ASD and has a ΔΔG of −1.01 kcal/mol (Figure 5C).

### 3.7. Evolutionarily Conserved Regions

Using Aminode, we found 50 region evolutionarily conserved regions across the NRXN1 protein, and the human NRXN1 AlphaFold model covers 48 of those completely and partially covers the 49th region. We evaluated the stability changes over the evolutionarily conserved regions. We found approximately 61% of the mutations generated in positions across the ECRs resulted in stability changes that were moderate to high. In comparison, 39% of mutations generated were found to have a neutral or moderately stabilizing effect. The stability heat map of positions corresponding to ECRs demonstrates that most of the positions where mutagenesis has fewer destabilizing effects are found toward the end of the protein model between positions 1303 and 1477. This region overlaps with the 4.1 m binding motif (1422–1440), the sequence of which is MYKYRNRDEGSYHVDESRN. Welch’s two-sample *T*-test resulted in a significant difference being found between the ΔΔG of evolutionarily conserved regions and nonconserved regions (t = −6.9189, *p*-value = 4.645 × 10^12^).

No correlations were found between mean ΔΔG as predicted by Foldx and Consurf normalized score. We performed a one-way ANOVA on normalized Consurf scores and mean ΔΔG grouping (i.e., highly destabilizing, moderately stabilizing, neutral, etc.). There were significant differences between the variances of these groups in terms of their Consurf scores per one-Way ANOVA (F = 3.02, Pr > 0.00746). Tukey test demonstrated that this difference was primarily found between highly conserved and highly variable residues (adj *p*-value < 0.01).

The conserved domain database identified seven conserved domains with significant domain-specific E-values [45]. These seven domains represented the Laminin G and 4.1 m superfamilies 1. Two additional domains were found: the EGF-like and the Syndecan-like domains; however, they failed to meet the domain-specific E-value threshold. The positions of the Laminin G and EGF conserved domains roughly mirror the positions of the recognized domains for the entire length of NRXN1 protein (Supplementary Figure S3). For example, the second EGF domain, which spans positions 676–713, is also a highly conserved domain, with an E-value of 2.982 × 10^−07^ (CDD). We compared the stability of conserved and nonconserved domains using one-way ANOVA (df = 9, F = 92.1, *p* = 2 × 10^−16^) and found there was a significant difference between the groups. The pairwise results of the Tukey test are displayed in Supplementary Table S1. Significant differences were found between the variances of the conserved Lam G1, LamG2, and LamG4 domains when compared to the conserved LamG3 domains (*p* < 0.01) (Supplementary Table S1).

## 4. Discussion

We have demonstrated the usefulness of AlphaFold models to investigate the evolution of proteins such as NRXN1. Using the AlphaFold structures and experimental structures in conjunction with bioinformatics tools, we can compare the structures and biophysical properties of proteins to better understand their function. The AlphaFold model is reliable for comparative analysis of homologous proteins. We can investigate and compare the effects of mutations on homologous proteins to aid our understanding of how mutations may affect protein structure and function and, therefore, how they may contribute to disease processes.

There are few studies to date that have utilized AlphaFold to investigate protein variations related to mental disorders. A previous study of a de novo heterozygous variant, V456A, in the RNA polymerase II subunit A (POLR2A) found that there were no gross structural deformities in the local structure when comparing the WT AlphaFold or mutagenized AlphaFold structures [47]. This variant was associated with intellectual and behavioral symptoms in a long-term case study of a patient who had also been diagnosed with ASD and epilepsy [47]. The author proposed that the weakening of hydrophobic bonds within the catalytic site was to blame for the deleterious effect of this mutation on the function of POLR2A protein [47]. Additionally, an investigation of the properties of yohimbine was performed in silico mutagenesis on an AlphaFold model of the 5HT receptor [48]. Several non-peer-reviewed studies have also utilized the AlphaFold to study specific mutations related to intellectual disability, neurodevelopmental and neurodegenerative disorders, and neuropsychiatric disorders [49,50,51,52].

Mental disorders such as SCZ and ASD and many neuropsychiatric symptoms are associated with mutations in NRXN1 and its binding partners. The majority of mutations we generated in positions associated with disease-causing mutations led to moderate to highly destabilizing changes in the overall free folding energy of the NRXN1 protein. One example is the L893V mutation that is associated with ASD. Although several of the disease-causing mutations themselves are moderately to highly destabilizing, there are some exceptions, including the highly stabilizing mutation H845Y, which is associated with ASD, and H1434R, which is associated with SCZ.

Few functional studies of NRXN1 disease-causing mutations can be found in the literature. For example, the L893V and I1135V mutations associated with ASD were found to decrease protein expression and disrupt circadian rhythms in transgenic flies when compared with control flies [53]. An in vitro study found reduced cell surface expression of NRXN1 and decreased binding of NRXN1 and NLGN1 for selected disease-causing missense mutations [54]. Neurexins seem to be important in the history of mammalian evolution, as mammals express three neurexin proteins, and invertebrates such as *Drosophila* and *C. elegans* only express NRXN1a [55]. Most evolutionary studies of NRXN1 have focused mainly on alternative splicing or the effect of mutations, as there is a large degree of structural and sequential similarity between mammalian and vertebrate neurexin proteins. However, due to the relative paucity of reliable protein models, these studies are limited to comparing biophysical tendencies or changes resulting from mutations that are limited in scope. Nevertheless, with better models, such as those generated with AlphaFold, we can better investigate more local structural and biophysical changes across species, which may reveal important evolutionary or functional insights [56,57]. We also demonstrated a significant correlation between the protein stability changes following alanine mutation across the AlphaFold and experimental NRXN1 models. Furthermore, we demonstrated significant differences in the mean effect of mutation on protein stability between evolutionarily constrained and nonconstrained regions of the AlphaFold NRXN1 model. These results demonstrate the utility of AlphaFold in exploring local structural and other differences in the evolutionary history of NRXN1 and other proteins.

There are, however, several limitations to utilizing this methodology. One limitation of using AlphaFold to study proteins involved in mental disorders is that AlphaFold may not adequately predict the conformations of intrinsically disordered regions of proteins [58]. As many as 80% of proteins currently investigated for their association with mental disorders include intrinsically disordered regions [59]. Additionally, modeling the relative positions of domains, modeling shifts in response to stimulation, etc., are issues that remain to be solved within ab initio protein structure modeling [60].

However, a recent study demonstrated that AlphaFold could reliably predict transmembrane proteins despite the fact that the algorithm’s training was based more on soluble proteins [61]. The study found that 53% of transmembrane regions had pLDDT scores above 90, indicating a high degree of confidence in the prediction [61]. Furthermore, we demonstrated how the transmembrane region prediction of AlphaFold aligns with the results of transmembrane domain prediction tools. Thus, while AlphaFold offers several advantages to the realm of structure modeling, there are several caveats one should keep in mind.

We have demonstrated how AlphaFold can be used to investigate evolutionary differences in proteins. Though we primarily investigated protein stability in the context of this paper, future work may interrogate the domain–domain interactions within proteins such as NRXN1 together with interactions with other proteins. It may be the case that some disease-causing variants may not have a significant effect on protein stability but might be important in the maintenance of the tertiary structure. The mutations we generated in several positions associated with deletion variants in the protein seem to have neutral to moderate effects on protein stability; however, their placement might make them important to maintaining the overall tertiary structure. An investigation of the domain–domain and protein–protein interactions might help explain how some mutations with modest effects on protein stability might still contribute to severe disease. This study provides a framework methodology for investigating aspects of protein evolution, including structure, protein stability, and conservation. The utility of this methodology extends beyond evolutionary research to the investigation of protein dynamics, disease-causing mutations, protein–protein interactions, and drug discovery.

## 5. Conclusions

We demonstrated that the human AlphaFold NRXN1 is a good model to use for studying protein structure and energetic changes resulting from missense mutations. Additionally, we demonstrated that AlphaFold can be used to compare homologous protein structures and their energetic landscapes. We found significant positive correlations when comparing the mean ΔΔG and ΔΔG of alanine mutations between the AlphaFold model and experimental structure. After analyzing 29,540 mutations across the entire human NRXN1 AlphaFold model, we determined the degree to which these mutations would affect protein stability. The vast majority of mutations we generated were moderately to highly destabilizing. We found roughly equivalent percentages for the bovine experimental structure, which shares 95.8% identity with the human sequence. Our analyses found several human NRXN1 mutations that are predicted to increase protein stability significantly: S341M, D539P, D951M, S871R, D951L (ΔΔG < −3.4 kcal/mol), and several that are predicted to decrease protein stability: G587W, G1096W, G850W, G1256W, and G182W (ΔΔG > 70 kcal/mol). Several of these mutations share equivalent positions in the *B. taurus* experimental structure. We also found that the majority of mutations we generated in evolutionarily conserved regions were predicted to be moderately to highly destabilizing. Therefore, we believe that AlphaFold, in conjunction with the use of experimental and homologous protein structures, can be extremely useful in investigating the effects of missense mutations on protein structure and for the investigation of protein evolution. 

## 6. Key Points

- We compared the human AlphaFold NRXN1 structure to the experimental bovine NRXN1 structure.
- The folding energy changes in the human AlphaFold model are highly corrected to those found for the bovine experimental structure.
- Several human NRXN1 mutations are predicted to affect protein stability significantly.
- The target mutations associated with brain disorders could destabilize human NRXN1 protein.

## Data Availability

Not applicable.

## Supplementary Materials

The following supporting information can be downloaded at: https://www.mdpi.com/article/10.3390/genes13050789/s1, Figure S1: Multiple Sequence alignment of NRXN1a protein sequences from 23 species; Figure S2: Structural alignment of SWISS-MODEL structure 3qcw.1.A with the bovine NRXN1 structure (PDB ID: 3poy). (B) Structural alignment of SWISS-MODEL structure 3qcw.1.A with the AlphaFold NRXN1 structure (Uniprot ID: Q9ULB1); Figure S3: Heatmap displaying the effects of saturation mutagenesis for the full-length human NRXN1 AlphaFold model (Top) and experimental bovine NRXN1 model (Bottom); Figure S4: Scatterplots depicting the corrections of ΔΔG of substitutions to 20 different residues between the AlphaFold and experimental structures; Figure S5: Heatmap of residues 691-702, the equivalent location of a consensus sequence for a hydroxylation site in homologous proteins; Figure S6: Transmembrane prediction from THMM (A), DeepTMHMM (B), and TOPCONS (C); Figure S7: Boxplot of SNAP2 predictions vs. stability prediction from FoldX; Table S1: Pairwise Results of Tukey Test comparing the DDG associated with mutations generated in residues in conserved domains and non-conserved regions of the NRXN1 AlphaFold structure; Table S2: Disease-causing NRXN1 mutations with DDG, Mean DDG, and SNAP2 scores from HGMD.
