# Supplementary material for: Computational Saturation Mutagenesis to Investigate the Effects of Neurexin-1 Mutations on AlphaFold Structure
Source: Genes (Basel). 2022 Apr 28;13(5):789. doi: 10.3390/genes13050789 (PMC9141173; doi:10.3390/genes13050789)
Supplement: Supplementary file 1 [file genes-13-00789-s001.zip › genes-1673742-supplementary.pdf]

genes MDPI

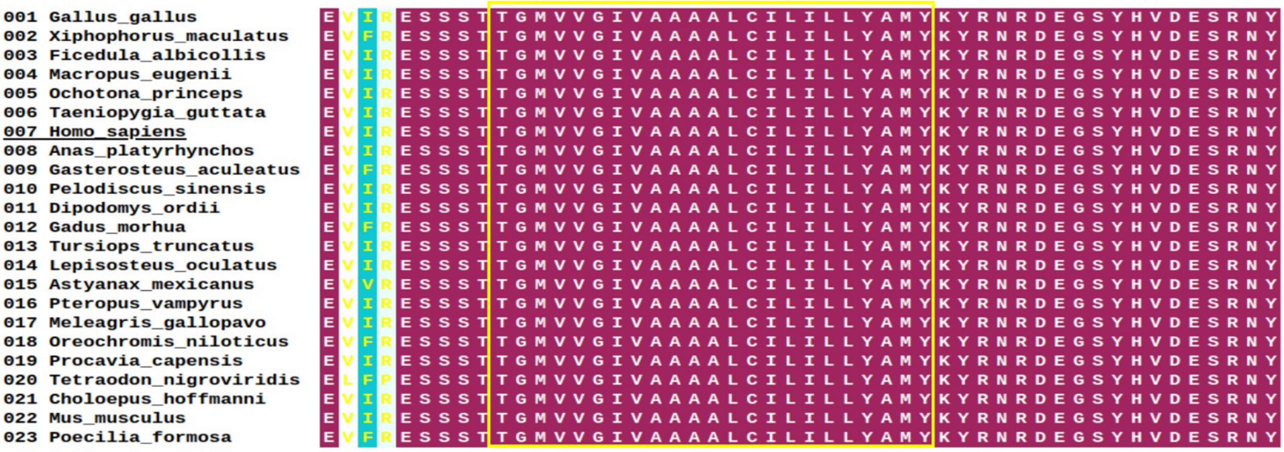

Supplementary Figure S1. Multiple Sequence alignment of NRXN1a protein sequences from 23 species. The yellow box highlights the region corresponding to the transmembrane region.

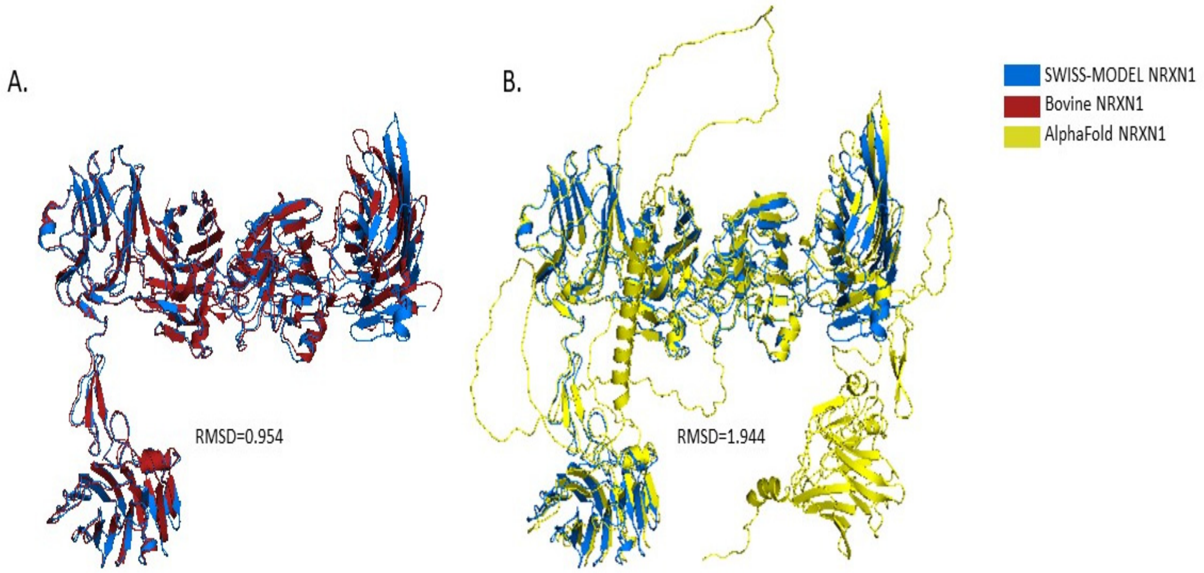

Supplementary Figure S2. (A) Structural alignment of SWISS-MODEL structure 3qcw.1.A with the bovine NRXN1 structure (PDB ID: 3poy). (B) Structural alignment of SWISS-MODEL structure 3qcw.1.A with the AlphaFold NRXN1 structure (Uniprot ID: Q9ULB1).

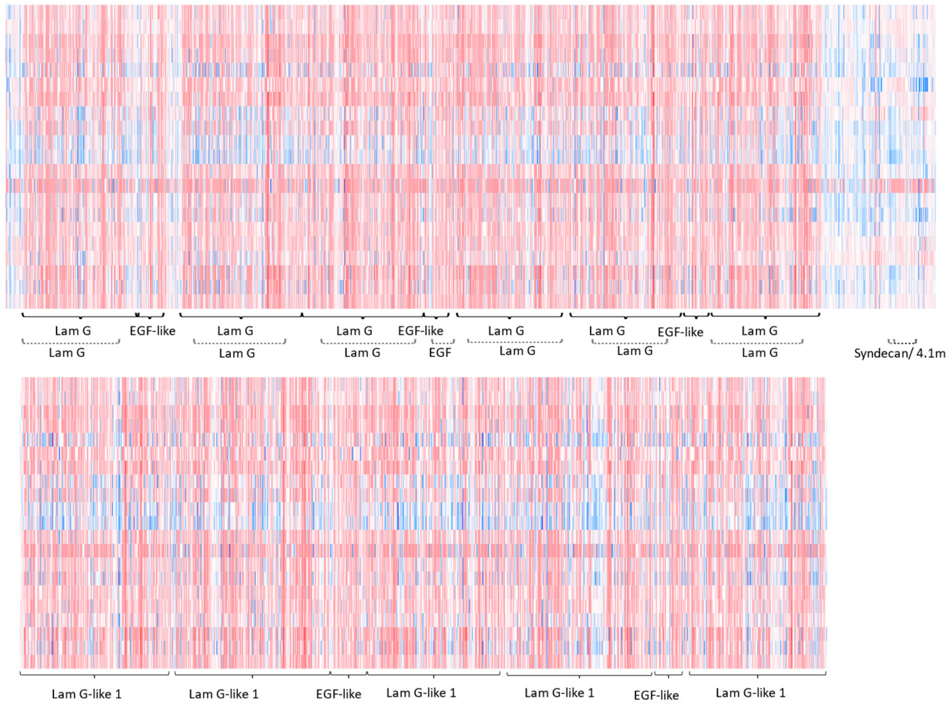

Supplementary Figure S3. Heatmap displaying the effects of saturation mutagenesis for the full-length human NRXN1 AlphaFold model (Top) and experimental bovine NRXN1 model (Bottom). The color gradient depicts the change in stability following mutagenesis: red indicates a decrease in protein stability, white represents a neutral change, and blue represents increased stability. The solid brackets show the relative locations of recognized functional domains. The dotted brackets indicate conserved domains as reported from the Conserved Domain Database.

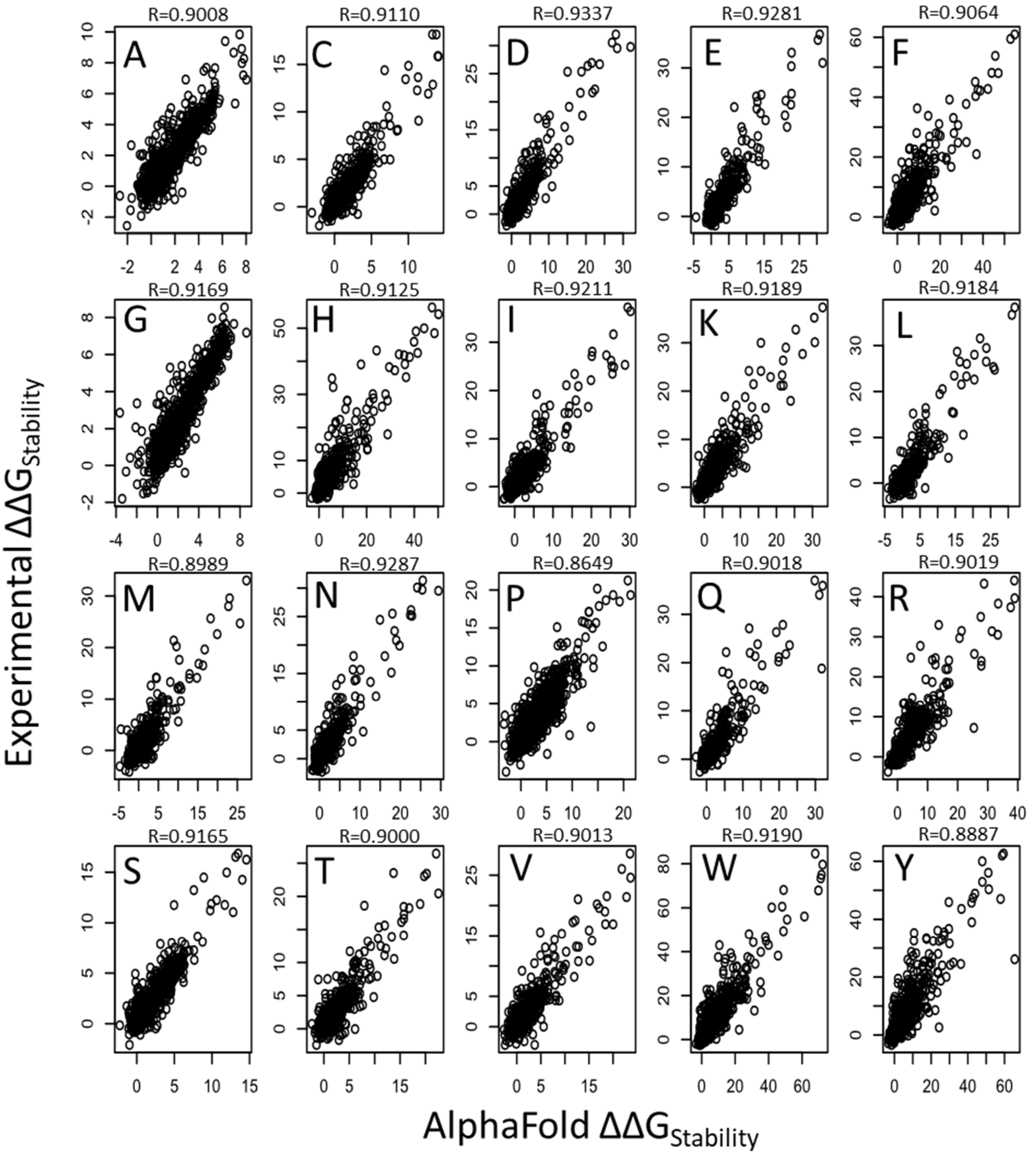

Supplementary Figure S4. Scatterplots depicting the corrections of ΔΔG of substitutions to 20 different residues between the AlphaFold and experimental structures.

	C691	R692	D693	G694	W695	N696	R697	Y698	V699	C700	D701	C702
A	2.903	1.953	1.342	1.798	3.251	2.1	1.751	1.023	1.507	4.065	3.027	2.919
C	-0.013	1.961	0.836	5.591	2.916	1.472	1.242	0.891	1.029	0	2.766	0.023
D	4.573	2.203	0	4.077	3.198	2.506	2.673	1.951	0.981	10.925	-0.012	4.793
E	3.586	1.647	0.204	6.448	3.902	2.884	2.323	1.21	0.697	9.325	2.42	4.179
F	2.657	0.618	0.434	3.913	0.169	2.334	1.4	-0.58	0.314	15.659	1.518	1.819
G	4.619	2.55	2.43	0	4.585	2.013	2.181	2.391	1.91	4.578	3.509	3.367
H	3.747	1.323	2.231	34.835	3.223	2.714	1.93	1.335	0.114	13.957	2.91	2.784
I	4.098	0.561	-0.146	6.048	4.114	3.953	1.671	2.827	-0.616	6.594	2.774	5.323
K	1.609	1.119	0.637	6.616	3.037	1.488	0.607	0.068	0.373	10.869	2.572	3.542
L	3.003	0.746	0.379	5.451	1.257	1.395	1.229	3.726	-0.212	6.526	2.15	5.767
M	2.229	0.465	-0.129	4.316	0.829	1.638	-0.225	0.018	0.282	6.565	2.385	2.801
N	3.871	1.971	0.77	3.545	3.105	-0.002	1.392	1.376	0.885	9.049	1.525	3.012
P	2.156	5.428	-0.956	7.132	3.204	6.338	7.619	-0.5	4.226	3.688	3.483	1.961
Q	3.253	1.363	0.964	5.99	3.394	1.741	0.9	0.784	0.776	8.092	2.898	4.021
R	2.645	-0.015	1.751	9.118	3.624	1.902	-0.23	0.668	0.841	9.482	2.731	3.495
S	4.152	2.211	1.494	2.11	2.452	1.719	1.54	2.145	2.082	5.141	3.483	3.275
T	4.313	1.863	1.255	6.476	1.4	1.871	1.386	2.542	1.326	7.923	3.39	7.503
V	4.852	1.544	0.327	10.448	3.387	4.627	1.968	1.952	0	5.339	2.745	5.896
W	3.201	1.293	0.751	5.485	-0.021	2.174	1.266	-0.201	-0.116	26.032	2.69	1.677
Y	2.736	0.637	0.78	6.169	1.304	2.387	1.634	-0.014	0.413	19.998	1.925	2.113
Mean	3.21	1.572	0.768	6.778	2.617	2.363	1.713	1.181	0.841	9.19	2.544	3.514

Supplementary Figure S5. Heatmap of residues 691-702, the equivalent location of a consensus sequence for a hydroxylation site in homologous proteins. The color gradient corresponds to the degree of stability change: dark red to light red indicates highly to moderately destabilizing mutations, white indicates neutral, and dark blue to light blue indicates highly to moderately stabilizing mutations. The ΔΔG values are displayed in each cell.

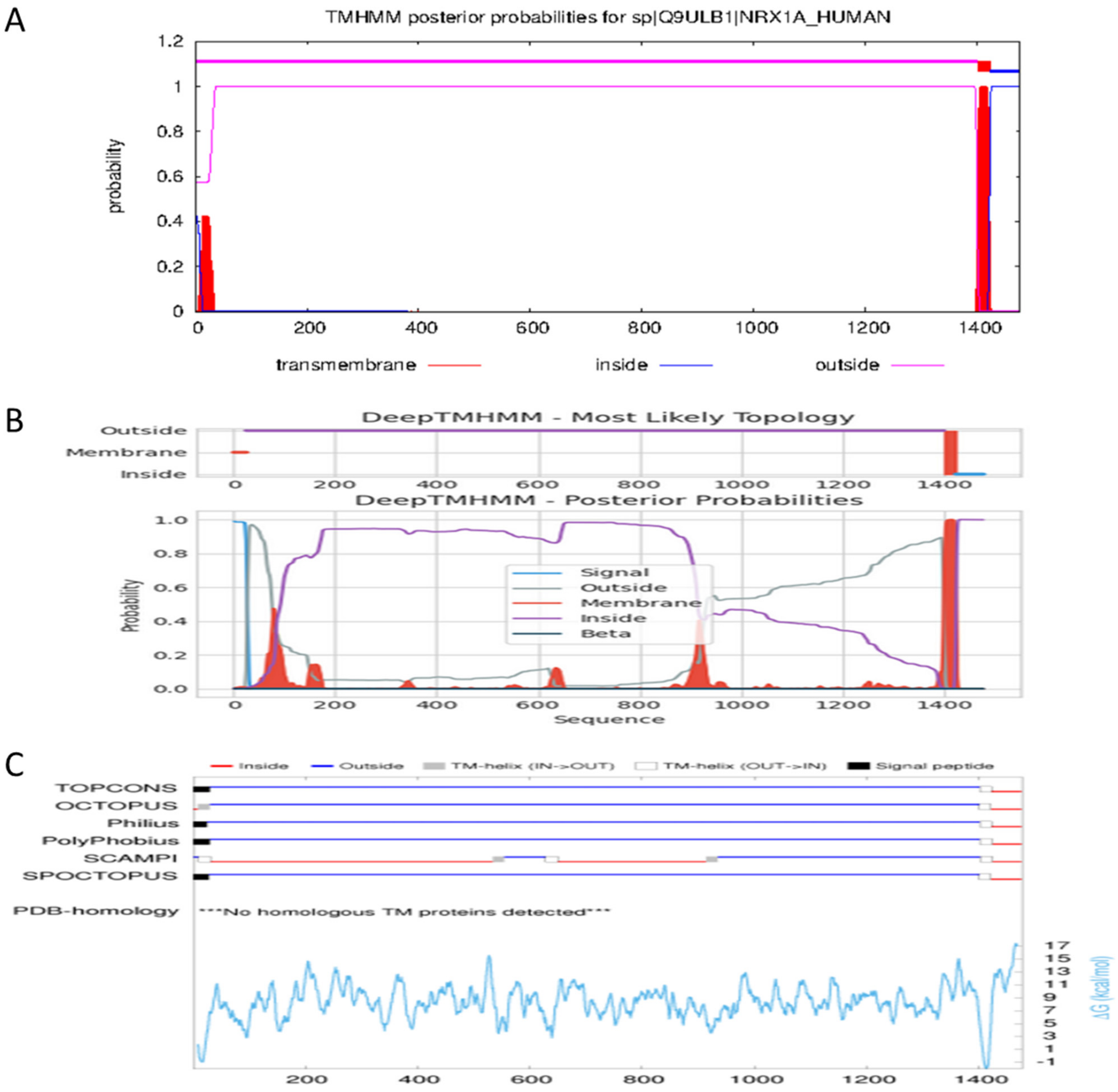

Supplementary Figure S6. Transmembrane prediction from THMM (A), DeepTMHMM (B), and TOPCONS (C). The sequence position is on the x axis. The vertical axes depict either probability or free folding energy

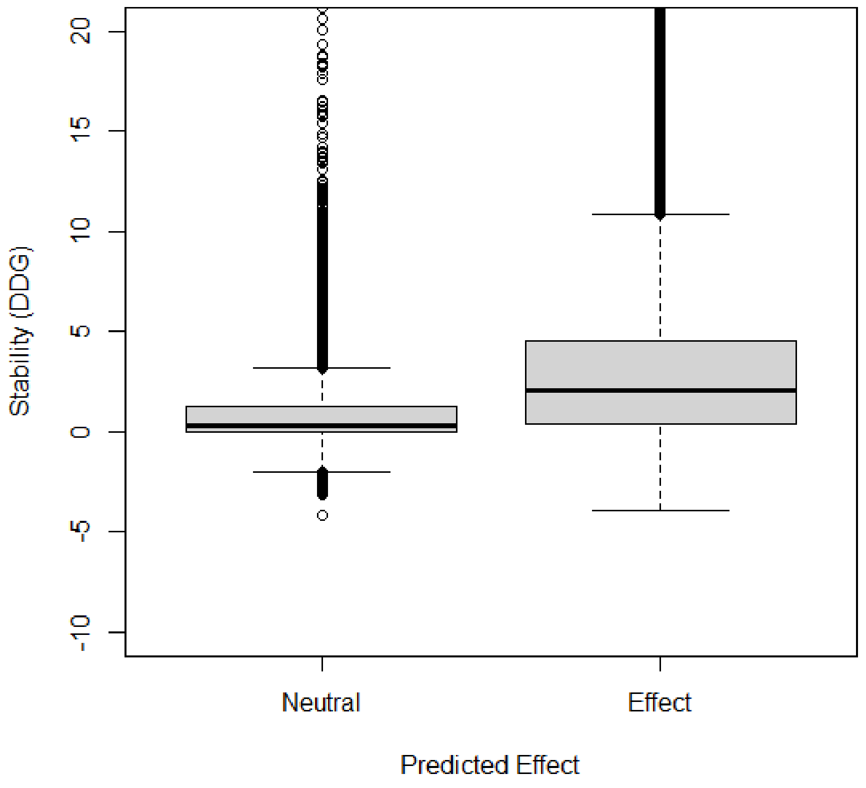

Supplementary Figure S7. Boxplot of SNAP2 predictions vs. stability prediction from FoldX. There was a significant difference between stability scores of mutations that are predicted to have an effect by SNAP2 and those predicted to be neutral.

Supplementary Table S1. Pairwise Results of Tukey Test comparing the DDG associated with mutations generated in residues in conserved domains and non-conserved regions of the NRXN1 AlphaFold structure.

	LamG1	LamG2	LamG3	EGF	LamG4	LamG5	LamG6	Syndecan	4.1m	Non-conserved
LamG1		1.00	0.00	0.40	0.89	0.99	0.88	0.00	0.00	0.00
LamG2	1.00		0.01	0.57	0.75	1.00	0.98	0.00	0.00	0.00
LamG3	0.00	0.01		1.00	0.00	0.10	0.24	0.00	0.00	0.00
EGF	0.40	0.57	1.00		0.05	0.85	0.94	0.00	0.00	0.00
LamG4	0.89	0.75	0.00	0.05		0.85	0.09	0.00	0.00	0.00
LamG5	0.99	1.00	0.10	0.85	0.32		1.00	0.00	0.00	0.00
LamG6	0.88	0.98	0.24	0.94	0.09	1.00		0.00	0.00	0.00
Syndecan	0.00	0.00	0.00	0.00	0.00	0.00	0.00		1.00	0.00
4.1m	0.00	0.00	0.00	0.00	0.00	0.00	0.00	1.00		0.00
Non-conserved	0.00	0.00	0.00	0.00	0.00	0.00	0.00	0.00	0.00	

Supplementary Table S2. Disease-causing NRXN1 mutations with DDG, Mean DDG, and SNAP2 scores from HGMD

Accession	DNA Mutation	Protein Variant	Phenotype	Reference	Source	DDG	Mean DDG	SNAP2
CM081373	c.53T>A	p.p.L18Q	Autism spectrum disorder	Kim (2008) Am J Hum Genet 82:199	PubMed 18179900 (NM_001135659.3)	0.24	0.45	neutral
CM186462	c.64G>C	p.p.G22R	Autism spectrum disorder	Takata (2018) Cell Rep 22:734	PubMed 29346770 (Transcript: ENST00000404971.5)	0.03	0.78	effect
CM1313262	c.455G>A	p.p.G152D	Autism spectrum disorder	Koshimizu (2013) PLoS One 8:e74167	PubMed 24066114 (NM_001135659)	1.53	2.23	neutral
CM1617422	c.587C>A	p.p.P196H	Autism spectrum disorder	Wang (2016) Nat Commun 7:13316	PubMed 27824329 (NM_001135659.1)	1.96	1.74	neutral
CM122179	c.943T>C	p.p.Y315H (Y282H)#	Autism	Liu (2012) J Psychiatr Res 46:630	PubMed 22405623 (Table 1)	1.62	2.16	neutral
CM1617273	c.1174A>C	p.p.N392H (N359H)#	Autism spectrum disorder	Wang (2016) Nat Commun 7:13316	PubMed 27824329 (NM_001135659.1)	0.38	0.1	neutral
CM081374	c.2242C>A	p.p.L748I (L708I)#	Autism spectrum disorder	Kim (2008) Am J Hum Genet 82:199	PubMed 18179900 (NM_001135659.3)	0.73	0.82	neutral
CM1819545	c.2354G>A	p.p.R785Q (R745Q)#	Autism spectrum disorder	Guo (2018) Mol Autism 9:64	PubMed 30564305 (NM_001135659)	1.58	3.29	effect
CM118317	c.2557C>T	p.p.R853C (R813C)#	Intellectual disability, nonsyndromic	Gauthier (2011) Hum Genet 130:563	PubMed 21424692 (Supplementary Table 1)	2.48	2.2	effect
CM137480	c.2653C>T	p.p.H885Y (H845Y)	Autism spectrum disorder	Jiang (2013) Am J Hum	PubMed 23849776 (NM_001135659.3)	-1.01	0.62	neutral

				Genet 93:249				
CM142515	c.2663A>G	p.p.E888G (E848G)#	Schizophrenia	Fromer (2014) Nature 506:179	PubMed 24463507 (Supplemtary Table 1)	4.13	4.74	effect
CM148697	c.2713T>A	p.p.F905I (F865I)#	Autism spectrum disorder & intellectual disability	Yangngam (2014) Genet Test Mol Biomarkers 18:510	PubMed 24832020 (Figure 1)	2.89	3.27	effect
CM122180	c.2797C>G	p.p.L933V (L893V)#	Autism	Liu (2012) J Psychiatr Res 46:630	PubMed 22405623 (Table 1)	4.14	2.01	neutral
CM122181	c.3523A>G	p.p.I1175V (I1135V) #	Autism	Liu (2012) J Psychiatr Res 46:630	PubMed 22405623 (Table 1)	1.25	5.59	neutral
CM1414132	c.3932G>T	p.p.R206L(R1241L) #	Nicotine dependence risk, association with	Yang (2015) Mol Psychiatry 20:1467	PubMed 25450229 (Table 2.)	-0.02	-0.06	effect
CM1414131	c.4135A>C	p.p.T274(T1309P) #	Nicotine dependence risk, association with	Yang (2015) Mol Psychiatry 20:1467	PubMed 25450229 (Table 3., rs77665267, NM_138735.5)	-0.15	-0.09	effect
CM118318	c.4511A>G	p.p.H1504R(H1434R) #	Schizophrenia	Gauthier (2011) Hum Genet 130:563	PubMed 21424692 (Figure 1)	-0.5	-0.18	neutral

#The mutation position in parentheses is aligned based on the canonicali sequence of human NRXN1a (UniProt ID: QU9LB1).
